# Connecting the dots: Network structures of internalizing and functional symptoms in a population-based cohort

**DOI:** 10.1016/j.jpsychores.2024.111932

**Published:** 2024-09-13

**Authors:** Urvi Saini, Judith G.M. Rosmalen, Albertine J. Oldehinkel, Hanna M. van Loo

**Affiliations:** a University of Groningen, University Medical Center Groningen, Department of Psychiatry, Interdisciplinary Center Psychopathology and Emotion Regulation (ICPE), Groningen, the Netherlands; b University Medical Center Groningen, Department of Internal Medicine, Groningen, the Netherlands

**Keywords:** Chronic fatigue syndrome, Fibromyalgia, Functional disorders, Internalizing disorders, Irritable bowel syndrome, Network analyses, Mental disorders

## Abstract

**Objective:**

Comorbidities between internalizing disorders (IDs) and functional disorders (FDs) are well-documented, indicating shared pathways. However, their symptom-level relationships have been largely unexplored. This exploratory study employs a network approach to investigate symptoms of major depressive disorder (MDD), generalized anxiety disorder (GAD), myalgic encephalomyelitis/chronic fatigue syndrome (ME/CFS), fibromyalgia (FM), and irritable bowel syndrome (IBS) to identify bridge symptoms explaining comorbidity between the two domains.

**Methods:**

We used cross-sectional data on 72,919 adult subjects from the Lifelines Cohort Study, a Dutch general population sample. A total of 38 symptoms representing diagnostic criteria of IDs and FDs were assessed with validated questionnaires. Network models were estimated using eLasso, based on the Ising model, to identify bridge symptoms. The Network Comparison Test (NCT) was used to test whether there were differences in network structure and strength across sex and age.

**Results:**

Symptoms were moderately connected, with a network density of 52.7%. ID and FD symptoms clustered in their respective domains, but were connected through the bridge symptoms, fatigue, difficulty concentrating, trouble sleeping, and unrefreshing sleep. Fatigue and difficulty concentrating had the most connections, associated with 86.6% and 78.9% of the other symptoms, respectively. NCTs indicated no differences in network connectivity between females versus males or younger versus older adults (>50 years).

**Conclusions:**

ID and FD symptoms are moderately interconnected. Bridge symptoms displaying strong connections to multiple disorders may play a central role in the mechanisms underpinning the comorbidity between IDs and FDs.

## 1. Introduction

Internalizing disorders (IDs) form a cluster of disorders that include, among other disorders, major depressive disorder (MDD) and generalized anxiety disorder (GAD) [1]. Functional disorders (FDs) are syndromes characterized by persistent somatic symptoms for which detectable pathophysiological abnormalities are currently not found [2]. The three most well-known FDs are myalgic encephalomyelitis/chronic fatigue syndrome (ME/CFS), fibromyalgia (FM), and irritable bowel syndrome (IBS). Both IDs and FDs can be disabling, difficult to treat, and put a large burden on the healthcare system [3-7].

FDs are highly comorbid with IDs, with overlapping symptoms and familial coaggregation [8-10]. Individuals with FDs, particularly those with ME/CFS, have a higher prevalence of mood and anxiety disorders than those without FDs (odds ratios = 3.9–12.6) [10]. Moreover, individuals with multiple FDs have a higher risk of comorbid mental disorders than those with a single FD [11]. IDs and FDs may share etiologic pathways based on their co-occurrence, but studies on these overlapping pathways are lacking. Studying the symptom-level relationship between IDs and FDs can help identify shared pathways and provide insights into their etiology.

Network analysis can explore symptom-level relationships within IDs and FDs and is based on the assumption of causally connected symptoms [12,13]. Due to the cross-sectional nature of our study, the model captures only partial associations. Therefore, we cannot draw conclusions regarding the direction and causal nature of the effects. Here we suggest that overlapping criteria may explain the high levels of comorbidity observed. Bridge symptoms, defined as symptoms belonging to the defining criteria of more than one disorder, may facilitate connections between the disorders by connecting the symptom domains [12]. Identifying bridge symptoms within the network can therefore shed light on comorbidity pathways, thereby contributing to a better understanding of potential intervention points.

No prior study has explored symptom-level associations between IDs and FDs in the general population. Studies examining symptom patterns between IDs and FDs have either focused on specific groups (e.g., only patients with ME/CFS) [14], limiting generalizability to other populations, or used symptoms that are not part of the diagnostic criteria for these disorders, thereby reducing the clinical relevance and ability to compare results across studies [15,16]. Furthermore, they use small sample sizes [17], while a robust sample size is necessary for reliably measuring connectivity across a larger group of symptoms, particularly when comparing relatively rare symptoms. Altogether, there is a need for large cohort studies assessing IDs and FDs at the symptom level, using the symptoms included in standardized diagnostic criteria [9,18,19].

Sex and age differences in the network structure of ID and FD symptoms remain unexplored, despite evidence suggesting different manifestation patterns across these groups. Interestingly, although IDs and FDs are more often seen in women, the comorbidity between the two domains has been found to be higher in men [10], which could indicate higher connectivity across ID and FD symptoms in men. Furthermore, despite previous research indicating no sex differences in the network structure of IDs [20,21], differences in coping mechanisms and symptom interpretation between men and women suggest potential differences in symptom connectivity [22-25]. Previous studies have described age-related differences in symptom profiles for IDs and FDs [26,27]. Given the differences in symptom endorsement across age [28-31], through a network analysis, we could assess if symptom networks of IDs and FDs evolve over the lifespan. Studying sex and age-related differences through network analysis could provide insights into how symptoms of these disorders manifest differently across these groups, informing tailored intervention strategies.

In this explorative study, we investigate networks of symptoms of two IDs (MDD and GAD) and three FDs (ME/CFS, FM, and IBS). Our aim is to identify the presence of bridge symptoms that account for a portion of the comorbidity between IDs and FDs, and investigate potential sex and age differences therein. Because of the high rates of comorbidity, we expect positive symptom correlations between the two internalizing disorders (MDD, GAD) and the three functional disorders (ME/CFS, FM, IBS), with overlapping symptoms (i.e., fatigue, difficulty concentrating, trouble sleeping, and unrefreshing sleep) being centrally positioned within the network structures.

## 2. Methods

### 2.1. Preregistration

The sample, included variables, measurements, and planned analyses were preregistered on June 26, 2023 on Open Science Framework prior to any data analysis [32]. Deviations from the preregistration are summarized in the Supplemental Methods.

### 2.2. Subjects

#### 2.2.1. Lifelines cohort study

All data were extracted from Lifelines. Lifelines is a multidisciplinary- prospective population-based cohort study examining in a unique three-generation design the health and health-related behaviors of 167,729 persons living in the North of the Netherlands [33]. It employs a broad range of investigative procedures in assessing the biomedical, socio-demographic, behavioral, physical and psychological factors which contribute to the health and disease of the general population, with a special focus on multi-morbidity and complex genetics. For a detailed description of Lifelines’ recruitment procedure, we refer to previous studies [33-35]. The Lifelines cohort study has been approved by the Medical Ethical Committee of the University Medical Center Groningen. All participants provided written informed consent for participation. Data of the Lifelines cohort study are kept confidential and are only used for medical research purposes.

#### 2.2.2. Participants

We used data from participants aged 18 years and older collected during the second assessment wave of Lifelines, between 2014 and 2017 (*N* = 124,330, response rate = 76%). For 72,919 adult participants, data on symptoms for all five IDs and FDs were available and included in this study.

### 2.3. Measurements

#### 2.3.1. Internalizing disorders

The Mini International Neuropsychiatric Interview (MINI) assessed symptoms of current major depressive disorder (MDD) during the past two weeks and symptoms of generalized anxiety disorder (GAD) during the past six months. The MINI is a brief, reliable and valid structured diagnostic interview used to assess common mental disorders as defined by the DSM-IV (Supplemental Methods) [36]. The following twelve MDD symptoms representing the symptom criteria for MDD in the DSM-5 were included: depressed mood, anhedonia, appetite change, weight gain, weight loss, trouble sleeping, psychomotor retardation, psychomotor agitation, fatigue, worthlessness/guilt, difficulty concentrating, and suicidal ideation. In the MINI, insomnia and hypersomnia were aggregated into a single symptom about sleeping troubles. Therefore, trouble sleeping is an aggregated symptom, while weight loss/gain and psychomotor retardation/agitation remained disaggregated. For GAD, the seven symptoms representing the symptom criteria for GAD in the DSM-5 were used: general worry/anxiety, restlessness, muscle tension, fatigue, difficulty concentrating, irritability, trouble sleeping.

#### 2.3.2. Functional disorders

Symptoms of ME/CFS were assessed using the 1994 Centers for Disease Control and Prevention (CDC) criteria [37]. CFS is diagnosed based on the following symptoms: fatigue, sore throat, tender lymph nodes, muscle pain, joint pain, headaches, unrefreshing sleep, post-exertional malaise, and impaired memory or impaired concentration. In Lifelines, impaired memory and impaired concentration were assessed by separate items, which were first dichotomized and then combined into one symptom (‘difficulty concentrating’). The combined symptom follows the CDC criteria, which require the presence of either impaired memory or concentration or both.

Symptoms of FM were assessed following the recommendations in the 2016 update of the 2010 American College of Rheumatology (ACR) criteria [38,39]. Participants were asked to indicate in which of the 19 stated body areas they had experienced pain during the last week, by using the Widespread Pain Index (WPI) [38]. Fatigue and cognitive symptoms were assessed using items from the Checklist Individual Strength (CIS) [40].

Symptoms of IBS were surveyed using the ROME III IBS Diagnostic Questionnaire (DQ) [41]. The criteria, including occurrence of symptoms, were later adjusted in accordance with the current ROME IV criteria [42]. To meet the diagnostic criteria for IBS, participants had to have recurrent abdominal pain or discomfort at least one day per week for at least 6 months, as well as at least two of the three additional symptoms (pain improvement with defecation, onset of pain/discomfort associated with a change in stool frequency, and onset of pain/discomfort associated with a change in stool appearance). The three additional symptoms are conditional on the presence of recurrent abdominal pain. If the participant does not experience abdominal pain, these additional symptoms are not assessed. Therefore, for the analysis, the IBS symptoms were combined into one binary variable, which was scored (1) if the participants reported recurrent abdominal pain or discomfort that was sometimes to always accompanied by at least 2 of the 3 additional symptoms; and (0) in all other cases.

All FD symptoms were dichotomized and completed as self-report questionnaires (Supplemental Methods).

### 2.4. Statistical methods

#### 2.4.1. Overlapping symptoms

Some symptoms are part of the symptom criteria of several IDs/FDs (i.e., fatigue is part of the criteria of all disorders except IBS). To prevent redundancy and mitigate multicollinearity, we inspected tetrachoric correlations between the overlapping symptoms and combined them into one item if correlations were sufficiently high (*r* > 0.50). The overlapping symptoms and the disorders they presented in were trouble sleeping (MDD, GAD), unrefreshing sleep (ME/CFS, FM), fatigue (MDD, GAD, ME/CFS, FM), and difficulty concentrating (MDD, GAD, ME/CFS, FM). Trouble sleeping and unrefreshing sleep were not combined, as they represent different aspects of sleep disturbance [43]. The WPI left-right items were combined (i.e., left hip and right hip combined into hip). For both overlapping and left-right items, if a symptom was reported as present in at least one item, the combined symptom was coded as 1 (present). IBS abdominal pain and WPI abdominal pain were not combined, as they measured different clinical features (Supplemental Methods). Tetrachoric correlations (r) were calculated to explore associations for the final set of symptoms using R package *psych* [44]. Correlations were interpreted as small (0 ≤ *r* ≤ 0.29), medium (0.30 ≤ *r* ≤ 0.49), or large (0.50 ≤ *r* ≤ 1.00) [45]. An overview of the final set of items can be found in Table 1.

#### 2.4.2. Network analysis

The network models for dichotomous variables were estimated using eLasso, based on the Ising model [46]. The following network estimates were calculated: 1) the adjacency matrix, representing the connection weights (*w*), and 2) network density (nd), which quantifies the extent to which nodes are interconnected, with values closer to 1 reflecting a denser network (Supplemental Methods). All analyses were performed using R version 4.2.1 [47]. Weighted networks of symptoms were fitted using the R package *IsingFit* (hyperparameter ɣ=0.25) [48] and visualized using the R package *qgraph* [49]. For clarity, only edge weights of ≥0.3 were visualized in the network.

#### 2.4.3. Missing data

Network analyses require datasets with no missing data. Therefore, we conducted multivariate imputation by chained equations using the package *mice* in R version 4.2.1 [50]. The missing data rate of the included sample was 11.3%. Imputation was done prior to symptom aggregation. Symptoms with missing values were included in the imputation: psychomotor agitation (MDD symptom), worry and muscle tension (GAD symptoms), and all FD symptoms. Missing values were imputed into five datasets, which were analyzed separately. The network results for the imputed datasets were pooled into one network structure by averaging the adjacency matrices. We acknowledge that averaging adjacency matrices may not accurately reflect the uncertainty introduced by the imputation process. Therefore, networks were also modeled on all five imputed datasets. Their network density was calculated and compared to that of the averaged network.

#### 2.4.4. Sex and age differences

To test for differences in symptom networks between subgroups, we compared networks using a Network Comparison Test (NCT). This permutation-based test assesses the difference between two networks by evaluating invariant network structure, invariant global strength, and invariant edge strength (Supplemental Methods). Sex and age group comparisons were conducted across all five imputed datasets using the *NetworkComparisonTest* package in R version 4.2.1 [51]. Previous studies report differences in ID and FD prevalence around the 50 years mark [30,31,52-54]. Therefore, to test for age differences in symptom connections, the sample was divided into two groups: young and middle-aged adults (≤ 50 years old, *n* = 39,250) versus older adults (> 50 years old, *n* = 33,669).

#### 2.4.5. Sensitivity analysis

We performed three sensitivity analyses, to assess (1) the effect of altering the severity threshold of IBS symptoms, (2) the effect of other medical conditions on the networks, and (3) the effect of unbalanced subgroup sizes. First, we repeated the network analysis by including participants with only abdominal pain rather than abdominal pain that meets the criteria for IBS. For this sensitivity analysis, abdominal pain was coded as 1 if it was present for at least one day per month. Second, we repeated the network analysis in participants without other somatic conditions with similar symptoms as the FDs. More specifically, we removed participants who self-reported the presence of rheumatoid arthritis, heart failure, ulcerative colitis, celiac disease, Crohn’s disease, or cancer (*n* = 2398). Participants with missing data for any of these conditions were also removed (*n*=5662). This analysis was done to ensure that, in the main network, the reported bodily symptoms were not due to other medical conditions. Lastly, the NCT subgroups based on sex and age were unbalanced in sample size, which may impact on network properties such as strength and density [55]. To determine whether results were sensitive to unequal sample sizes, we repeated the NCT with balanced samples. This was done by taking a random sub-sample of the larger group equal to the size of the smaller group. This was repeated 10 times to prevent sampling bias.

## 3. Results

### 3.1. Characteristics of the study population

A total of 72,919 subjects (59.8% female) were included in the analysis. Their average age was 49.2 years (SD = 12.4). Frequencies of the symptoms included in the network analyses are presented in Table 1. Symptom prevalence was significantly higher in females than in males for majority of the symptoms (Supplemental Table 1). Adults older than 50 years (M_age_=59.6, SD=7.3) reported fewer symptoms of MDD and GAD than adults 50 years or younger (M_age_=40.2, SD = 8.1). This was different for the FD symptoms: older adults reported more often pain symptoms (i.e., muscle pain, WPI items), whereas younger adults reported more often the IBS symptom, for example (Supplemental Table 1). Symptoms of MDD and GAD showed stronger tetrachoric correlations, both within and between disorders, than the symptoms of FDs (Supplemental Fig. 1).

### 3.2. Network structure

**Fig. 1.**
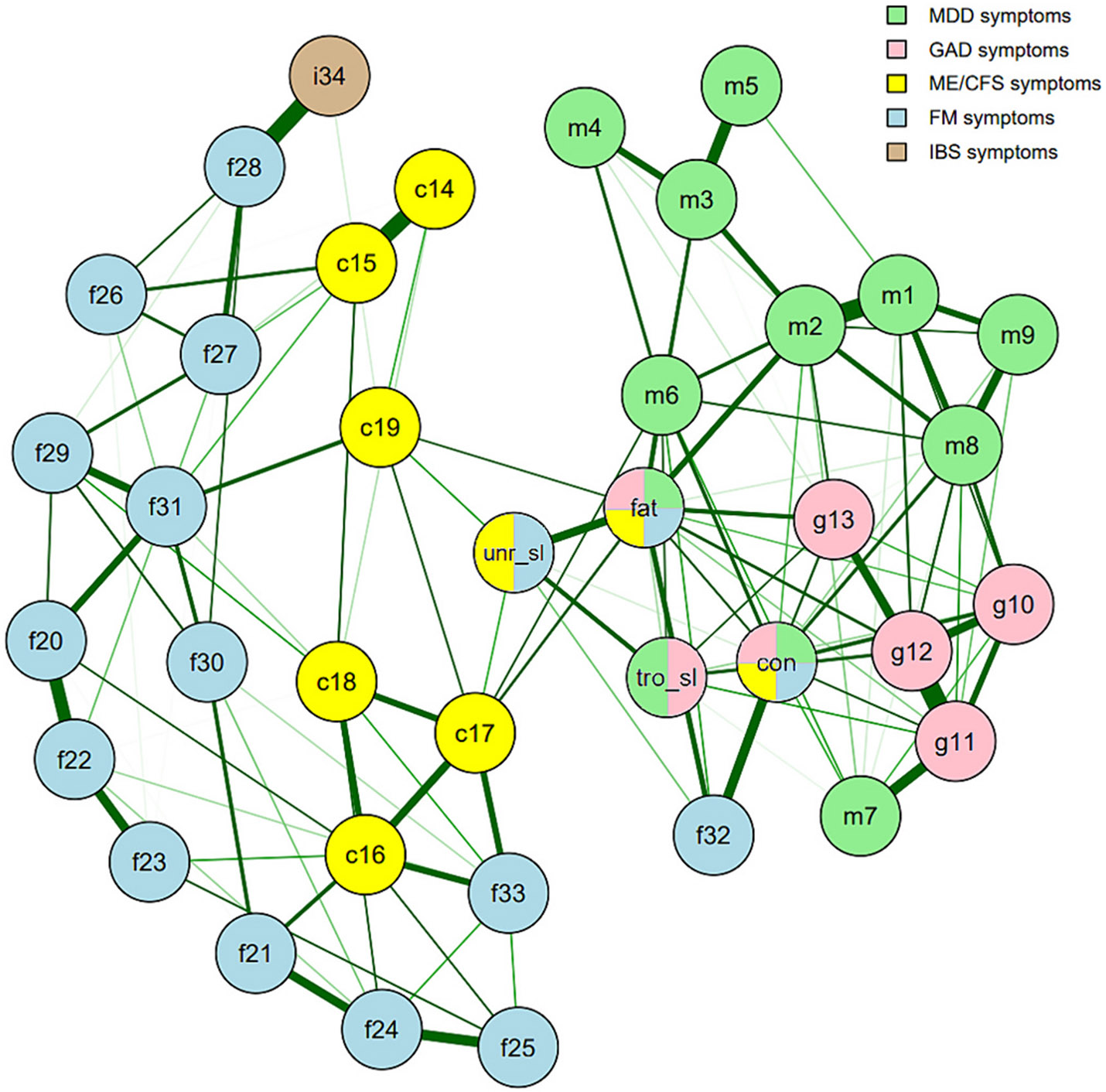
Full network as estimated with eLasso, in 72,919 individuals. Green lines indicate positive edges. Line thickness and color intensity display edge strength. Only edges with weight ≥ 0.3 are presented. Networks were fitted using the R package *IsingFit* (van Borkulo et al., 2016) and network plots were generated using the *qgraph* package (Epskamp et al., 2012). m1 = depressed mood; m2 = anhedonia; m3 = appetite change; m4 = weight gain; m5 = weight loss; m6 = psychomotor retardation; m7 = psychomotor agitation; m8 = guilt; m9 = suicidal; g10 = worry; g11 = restless; g12 = muscle tension; g13 = ir-ritability; c14 = sore throat; c15 = tender lymph nodes; c16 = joint pain; c17 = post-exertional malaise; c18 = muscle pain; c19 = headaches; f20 = WPI shoulder pain; f21 = WPI hip pain; f22 = WPI upper arm pain; f23 = WPI lower arm pain; f24 = WPI upper leg pain; f25 = WPI lower leg pain; f26 = WPI jaw pain; f27 = WPI chest pain; f28 = WPI abdominal pain; f29 = WPI upper back pain; f30 = WPI lower back pain; f31 = WPI neck pain; f32 = CIS difficulty thinking; f33 = musculoskeletal pain; i34 = IBS abdominal pain; tro_sl = trouble sleeping; fat = fatigue; con = concentration; unr_sl = unrefreshing sleep.

**Fig. 2.**
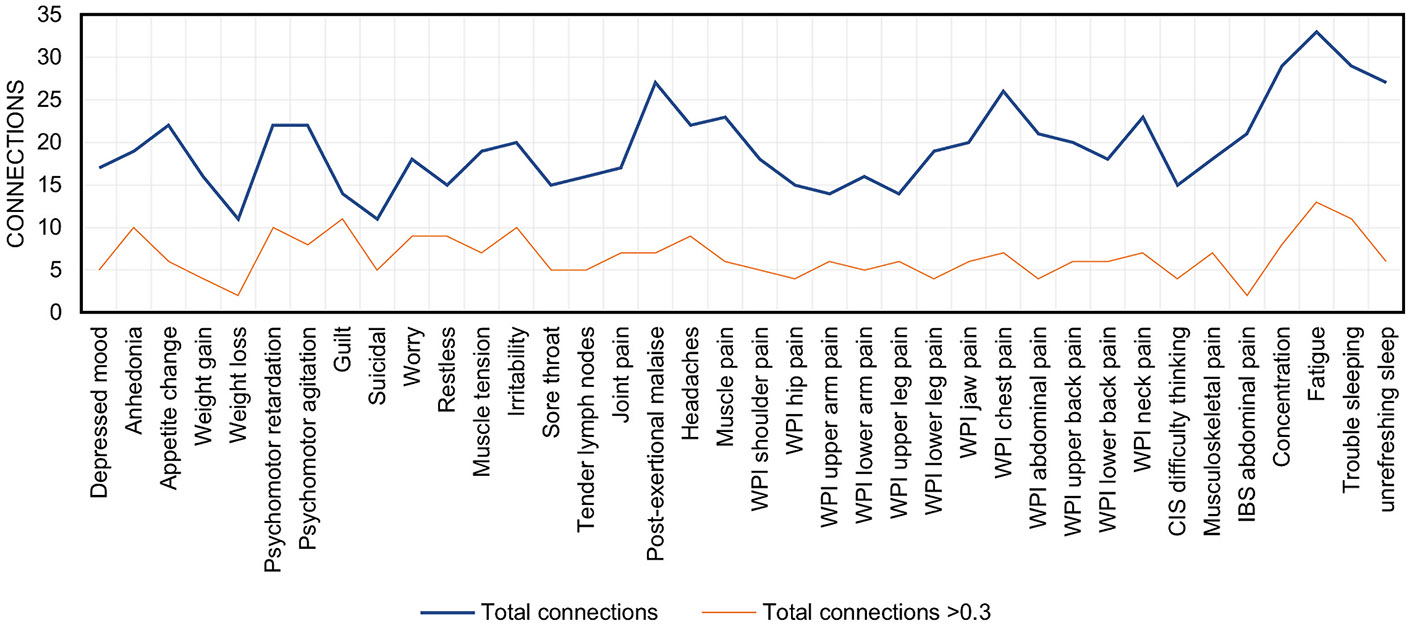
Number of connections per symptom. MDD, major depressive disorder; GAD, generalized anxiety disorder; ME/CFS, chronic fatigue syndrome; WPI, widespread pain index; CIS, checklist individual strength; FM, fibromyalgia; IBS, irritable bowel syndrome. WPI and CIS belong to the FM symptoms.

The symptom network of all participants was moderately connected (nd=0.53; Fig. 1). All edges in the network were positive. The ID domain was more connected (nd=0.78) than the FD domain (nd = 0.65). Difficulty thinking was the only non-overlapping FD symptom found in the ID domain of the network structure. The overall strongest connections were observed between GAD symptoms restlessness and muscle tension (*r* = 0.91; *w* = 1.0) and between MDD symptoms depressed mood and anhedonia (*r* = 0.89; *w*=0.95). Fatigue, concentration problems, and trouble sleeping presented with the greatest number of connections, ranging from 29 to 33 (Fig. 2). In general, the overlapping symptoms had the most connections: fatigue with 86.8%, concentration problems with 76.3%, trouble sleeping with 76.3%, and unrefreshing sleep with 71.1% of all other symptoms. Weight loss, suicidality, and upper arm pain were the least connected symptoms in the network. The ME/CFS symptoms joint pain and post-exertional malaise were strongly connected with the main symptom of FM, musculoskeletal pain (*r*=0.65; *w*=0.39 and *r*=0.68; *w*=0.38, respectively). Sore throat and tender lymph nodes were strongly connected to each other (*r*=0.63; *w*=0.82) but were weakly connected with the rest of the ME/CFS symptoms. IBS was moderately associated (*r* = 0.38) but highly connected (*w* = 0.86) with abdominal pain from FM, indicating that adjusting for other associations in the network affected the association between the two symptoms relatively less than most other associations. IBS showed no strong relationships with any of the other symptoms in the network. The network densities of all five imputed datasets were comparable to the network based on the averaged adjacency matrices (Supplemental Fig. 2).

The sensitivity analysis indicated that lowering the severity threshold for the IBS symptom (i.e., including participants with just abdominal pain instead of abdominal pain that meets the criteria for IBS) resulted into a comparable network structure (nd = 0.53; Supplemental Fig. 3). We repeated the network analysis in participants without a diagnosis of a medical condition that may present with similar symptoms as FDs (*n* = 64,859, mean age 49.1 (SD = 12.4), 60.2% females). The connectivity was comparable to that of the original network (nd = 0.51; Supplemental Fig. 4).

### 3.3. Sex and age-specific networks

The network structures of males (*n*=29,298) and females (*n*=43,621) separately are presented in Supplemental Fig. 5. The symptom network of the female subgroup was slightly more connected (nd = 0.50) than the network of the male subgroup (nd = 0.45), but this difference was not significant. The network structure of the two groups differed significantly in four out of the five imputed datasets, while the global strength was not significantly different for any (Supplemental Table 2). However, this difference in network structure became less consistent when the analysis was repeated with equally-sized subsamples (*n*=29,298), suggesting that the differences found in the primary analysis may be due to differences in power because of the unequal subsamples (Supplemental Table 3).

The network structures of younger adults (≤ 50 years) (*n*=39,250) and older adults (>50 years, *n*=33,669) are presented in Supplemental Fig. 6. The network of younger adults (≤ 50 years) was somewhat denser (nd=0.50) than that of the older adults (nd=0.46), but this difference was not significant. The network structure significantly differed between the two groups across all five imputed datasets, while no significant group differences in global strength were found across any of these datasets (Supplemental Table 2). Exploratory post-hoc testing of all edges indicated 81 edges that differed significantly between younger and older groups. Symptoms most often observed in these differences were upper and lower back pain, fatigue, musculoskeletal pain, trouble sleeping, restlessness, irritability, and psychomotor retardation. The *p*-values were not adjusted for multiple testing, as this is not necessary in an exploratory setting [55]. Therefore, the findings should be interpreted as exploratory, not as individual hypothesis tests of differences in the strengths of the associations. The sensitivity analysis using equally-sized subsamples (*n* = 33,669) did not show consistent differences in network structure or strength across all imputed datasets (Supplemental Table 4), suggesting that part of the differences found in the main analyses could be attributable to differences in sample size.

### 3.4. Post-hoc analyses

A diagnosis of FM is based on one core symptom, musculoskeletal pain, which is further detailed through specific WPI items. The specification of musculoskeletal pain in FM could strongly influence the symptom network, potentially overshadowing other relationships. Therefore, in addition to the planned network analysis, post-hoc analyses were conducted to explore the influence of the musculoskeletal pain and WPI items on the network (Supplemental Results). After removing the FM musculoskeletal pain item, the network (nd = 0.54) was comparable to that of the original network (Supplemental Fig. 7). Combining the WPI items increased the network density (nd = 0.63), indicating that these items may have dominated other symptom relationships in the network, especially those unrelated to pain (Supplemental Fig. 8).

## 4. Discussion

### 4.1. Summary of key findings

To our knowledge, this is the first large population-based study that examined the symptom network of two common IDs and three common FDs. Fatigue, difficulty concentrating, trouble sleeping, and unrefreshing sleep, all part of the diagnostic criteria of multiple disorders, were centrally positioned within the network structure, connecting the IDs and FDs. Together, these symptoms are hereafter referred to as the bridge symptoms. These bridge symptoms also had the highest number of connections within the network. Weight loss and suicidality were the symptoms with the lowest number of connections to other symptoms. Stronger connections were observed between MDD and GAD symptoms than between the symptoms of FDs. The IBS symptom abdominal pain was highly connected to abdominal pain in FM; however, it was not strongly associated with any other symptom. No differences in overall network connectivity were observed in sex and age comparisons.

### 4.2. Strengths and limitations of the study

The main strength of our study is the concurrent examination of the symptoms of IDs and FDs, assessed according to their diagnostic criteria, in a large general population cohort. Modeling the symptoms in a network structure captured their complex interrelationships. This study also has several limitations. First, the network is based on cross-sectional data and hence does not allow for determining the direction of the effects the symptoms may have on each other. Second, the network estimation technique eLasso demonstrates high specificity but moderate sensitivity [46]. While this suggests that the reported connections are likely accurate, it also indicates that some subtle connections may have been overlooked. Third, Lifelines consists of data of participants living in the north of the Netherlands, who are predominantly native Dutch. Symptoms of IDs and FDs may vary across cultures [56,57]. Therefore, the network structure may not be an accurate representation of symptom connectivity in other populations.

### 4.3. Symptom patterns and comorbidity

The symptoms forming two groups of MDD/GAD symptoms and functional somatic symptoms is consistent with previous studies examining MDD, GAD, and somatic symptoms [15,16]. Similar to our results, the ID and FD domains were connected through the bridge symptoms [15]. Difficulty thinking was the only non-overlapping FM symptom with connections to MDD and GAD. This may be possibly due to its conceptual overlap with a related symptom, concentration difficulty. This underscores the limited discriminative power of difficulty thinking in differentiating between FM and the other disorders. Notably, the connections between the bridge symptoms and other FD symptoms were weak; bridge symptoms were more strongly associated with MDD and GAD. One possible explanation is the stronger psychological interconnectedness between bridge symptoms (i.e., fatigue, difficulty concentrating, unrefreshing sleep) and MDD/GAD symptoms, potentially stemming from shared cognitive or emotional processes.

The FD pain symptoms did not have any strong connections with majority of the MDD symptoms. These connections fell below the threshold of 0.3 and were therefore not shown in the network. Network models use partial correlations, which control for the influence of all other symptoms in the network. Therefore, it is possible that the associations between pain and MDD symptoms may be due to their shared relationships with other symptoms in the network (e.g., pain ➔ fatigue ➔ depressed mood). Further research is necessary to study the direction of these relationships.”

The bridge symptoms are part of the diagnostic criteria for both IDs and FDs, leading to the possibility that their central positioning in the network may reflect this overlap. However, the population-based nature of our study makes this possibility unlikely: the study participants were not selected based on the presence or absence of specific disorders. Hence, the network analysis captured how symptoms naturally co-occur and interact in the general population, rather than in a selected subset of subjects satisfying disease criteria. It thus reveals how the bridge symptoms are connected to the other symptoms of different types of disorders.

Symptoms of ME/CFS, FM, and IBS differed in the number and strength of their connections. The high degree of interconnectivity between ME/CFS and FM symptoms, which was stronger when we aggregated the widespread pain symptoms, implies a potential underlying common mechanism between these conditions. This not only challenges the notion of distinct domains for ME/CFS and FM but also supports the consideration of a unified approach in understanding their underlying mechanisms. The minimal connections and weak associations observed between the IBS symptom abdominal pain and other symptoms in the network align with previous studies, highlighting a distinct comorbidity pattern for IBS within both the FD and ID domains [10,58].

The network highlights post-exertional malaise as a distinguishing feature between ME/CFS and FM, showing high connectivity with core symptoms of both disorders while maintaining a distinct pattern by weakly connecting with widespread pain. These findings support including post-exertional malaise as a required diagnostic criterion for ME/CFS, in agreement with more recently established criteria such as the Institute of Medicine criteria [59], the Canadian Consensus Criteria and the International Consensus Criteria [60,61]. Furthermore, these results suggest a difference in the discriminatory power of the two symptoms of FM, musculoskeletal pain and widespread pain. This is not surprising as the WPI is a detailed measure of musculoskeletal pain distribution across different body parts. The variations in diagnostic criteria underscore the challenges posed by the heterogeneity of ME/CFS symptoms and the ongoing refinement of our understanding of this disorder.

Our findings have potential implications for the classification of IDs and FDs. The distinct clusters of ID vs FD symptoms show that, at least in terms of symptoms, the current classification seems to effectively capture different type of syndromes. Within-domain symptom connections suggest that there is substantial symptom overlap between subjects with MDD and GAD, and between subjects with CFS and FM. However, symptoms alone do not fully capture the complexity required for accurate classification. A more useful classification should include information on other validators, such as shared etiology, reaction to treatment, and course of illness [62].

### 4.4. Network similarities across sex and age

In line with previous studies [11,63], ID and FD symptoms were more common in women than in men. However, our findings did not reveal any sex differences in symptom connectivity. In other words, while women may exhibit symptoms more frequently than men, their interconnections within the symptom network do not differ substantially. Due to a lack of comparable studies, it is difficult to compare our results with existing research. However, consistent with our findings, previous research found no sex differences in depressive symptom networks within individuals meeting MDD diagnostic criteria [20,21], nor in ME/CFS symptom patterns [64].

To our knowledge, no previous studies have simultaneously assessed the influence of age on the network structures of IDs and FDs. Although the network structure differed between younger and older adults, the symptom connectivity was comparable. It is possible that although the symptom connectivity is similar, symptom severity may differ between the two age groups. For example, a study on ME/CFS symptoms found a significant increase in fatigue severity in adults over 50 years of age [30]. Additional research considering symptom severity across different age subgroups could enhance understanding of age-related differences in ID and FD symptom patterns.

Consistent with a previous study that performed network comparisons [20], our sensitivity analysis suggested that NCT results may be influenced by unbalanced subgroups. Our results are also consistent with the results of an NCT validation study, which reported that statistical power decreases when sample sizes are unequal [51].

### 4.5. Future research

To better understand the mechanisms driving the comorbidity of these disorders, we recommend that future studies explore the development of symptoms over time. A longitudinal network analysis may reveal how symptoms influence each other and the order in which they develop over time. A previous study found that depressive symptoms with strong network connections more strongly predicted the onset of full-blown MDD than those with few or weak connections [63]. This suggests that bridge symptoms displaying strong connections to other domains may play a central role in the mechanisms underpinning the comorbidity between IDs and FDs. Consequently, these bridge symptoms emerge as potential targets for intervention, given their role in connecting the domains. As the present study looked at symptoms in the general population, it may be useful to repeat the analyses for persons that meet the diagnostic criteria of the disorders. Understanding symptom patterns in a clinical sample may further support clinicians in the development of targeted interventions.

## 5. Conclusion

This study examined ID and FD symptom patterns and identified several bridge symptoms connecting the two domains of disorders, and these were consistent across sex and age. The identified bridge symptoms may contribute to the high rates of comorbidity seen between IDs and FDs and are potential targets for interventions.

## Supplementary Material

Supplementary material

## Figures and Tables

**Table 1:** Frequency of diagnostic symptoms of internalizing disorders and functional disorders in study population.

Symptom	%	Symptom	%
**MDD**		**FM**	
Depressed mood	3.9	Musculoskeletal pain	14.8
Anhedonia	5.1	Shoulder pain	31.1
Appetite change	4.0	Hip pain	19.5
Weight gain	4.3	Upper arm pain	16.7
Weight loss	2.1	Lower arm pain	10.6
Psychomotor retardation	1.0	Upper leg pain	12.2
Psychomotor agitation	4.4	Lower leg pain	13.2
Guilt	3.0	Jaw pain	5.0
Suicidal	1.0	Chest pain	7.0
**GAD**		Abdominal pain	13.8
Worry	6.3	Upper back pain	13.1
Restless	15.6	Lower back pain	41.4
Muscle tension	24.5	Neck pain	32.2
Irritability	14.1	Difficulty thinking	14.1
**ME/CFS**		**IBS**	
Sore throat	1.6	IBS symptom	8.2
Tender lymph nodes	1.2	**Overlapping Symptoms**	
Joint pain	20.9	Trouble sleeping	31.5
Post exertional malaise	10.6	Unrefreshing sleep	37.9
Muscle pain	14.1	Fatigue	39.1
Headaches	7.3	Difficulty concentrating	32.5

## Data Availability

Data may be obtained from a third party and are not publicly available. Researchers can apply to use the Lifelines data used in this study. More information about how to request Lifelines data and the conditions of use can be found on their website (https://www.lifelines-biobank.com/researchers/working-with-us).

## Appendix A. Supplementary data

Supplementary data to this article can be found online at https://doi.org/10.1016/j.jpsychores.2024.111932.
