# Supplementary material for: Connecting the dots: Network structures of internalizing and functional symptoms in a population-based cohort
Source: J Psychosom Res. Author manuscript; Available in PMC 2025 Sep 22. (PMC12451014; doi:10.1016/j.jpsychores.2024.111932)
Supplement: Supplementary material [file NIHMS2110008-supplement-Supplementary_material.docx]

**Supplementary materials**

Table of Contents

**Supplemental methods2**

Preregistration deviations2

Symptom dichotomization2

Overlapping symptoms2

Network estimates3

NCT for sex and age differences3

**Supplemental results3**

Post-hoc analysis3

Supplemental Table 14

Supplemental Figure 16

Supplemental Figure 27

Supplemental Figure 3 9

Supplemental Figure 4 10

Supplemental Table 2 11

Supplemental Table 3 11

Supplemental Figure 5 12

Supplemental Table 4 13

Supplemental Figure 6 14

Supplemental Figure 7 15

Supplemental Figure 8 16

References 17

**Supplemental Methods**

*Preregistration deviations*

For FM, the somatic symptoms assessed using the 12-item somatization scale of the Symptom Checklist-90 (SCL-90 SOM)[1] were not included in the network analysis. The somatic symptoms measured by the SCL are only a few of the possible symptoms a participant may experience for a FM diagnosis. Therefore, only the symptoms specifically part of the diagnostic criteria were assessed.

In the final analysis, we did not compute the centrality indices for the networks. This was due to the ongoing debate regarding their interpretation in psychometric network models [2]. Additionally, the lack of clear guidelines for interpreting various centrality indices would further complicate their applicability in our exploratory context.

In the preregistration, we planned to conduct a sensitivity analysis to examine the effect of symptom duration and severity on the network model. This analysis was not performed due to inconsistencies in the measurement scales across questionnaires. While some questionnaires provided a detailed scale for symptom frequency (e.g., CFS symptoms assessed using a 1-4 scale), others used only a yes/no format (e.g., MINI and WPI), which prevented a meaningful assessment of the impact of symptom duration and severity on the network model.

*Symptom dichotomization*

MINI: all symptoms were binary items, and assessed with the probe “during the past 2 weeks…”. Questions were answered in a yes/no format with yes being coded as 1 and no as 0. There were no skips in the data, therefore participants were asked to answer all items. The MINI was administered as a digital questionnaire on site.

CFS symptoms measured using the CDC criteria: Participants rated the symptoms on a scale of 1 to 4 (1 = “not at all”; 4 = “every day”). To dichotomize data, for all items, a score of 1 and 2 were coded as 0 and scores of 3 and 4 were coded as 1. If either impaired memory or impaired concentration was 1, then the combined symptom was 1 and if both were 0, then the combined symptom was 0. If either impaired memory or concentration were NA, then the combined symptom was NA if the other was NA or 0, and 1 if the other was 1.

Checklist Individual Strength (CIS): on a scale of 1 (“yes, true”) to 7 (“no, not true”), participants were asked to rate whether, in the last two weeks in general, they felt tired, had difficulty thinking, took an effort to concentrate, and did not wake up rested. To dichotomize the data, a score between 1 to 3 was coded as 1 and a score between 4 to 7 was coded as 0.

CFS and IBS symptoms were assessed with the probe “during the past 6 months…”. For FM, the WPI items were assessed within a seven-day frame, musculoskeletal pain in the past 6 months, and CIS items within a two weeks period

*Overlapping symptoms*

Our decision to keep abdominal pain for FM and IBS separate was based on them representing different clinical features within each disorder. The abdominal pain symptom in IBS is more comprehensive, as it includes additional criteria such as pain improvement with defecation, onset of pain associated with a change in stool frequency, and onset of pain associated with a change in stool appearance. In contrast, the abdominal pain symptom for FM, as measured by the WPI, is more general, as it measures pain in that region in a yes/no format only. Combining these symptoms could therefore mask important differences in their role within the network model.

*Network estimates*

Network density represents the proportion of connections that exist in the network relative to the total possible connections. The closer the value is to 1, the denser the network is. Adjacency matrix indicates which nodes are connected and the strength of their connections. To scale the matrix into a [0,1] range, weights (*w*) were normalized using the Min-Max Normalization method (Han, Kamber, & Pei, 2012). Weight strengths were interpreted using the same ranges as the correlations (Cohen, 2013).

*NCT for sex and age differences*

NCT is a permutation-based test which assesses the difference between two networks by evaluating three hypotheses. These hypotheses test: (1) invariant network structure, which states that the structure is identical across subgroups; (2) invariant global strength, which states that, although networks may differ in structure, the overall level of connectivity is similar across groups; and (3) invariant edge strength, which focuses on the difference in strength of specific edges. Test of edge invariance was applied as a post-hoc analysis if the network invariance test was significant (van Borkulo et al., 2023). Post-hoc testing on significant edges were exploratory and hence the p-values were not corrected for multiple testing (van Borkulo et al., 2023).

**Supplemental Results**

*Post-hoc analysis*

The diagnostic criteria for MDD, GAD, and CFS share similarities in requiring one or two core symptoms along with additional symptoms for diagnosis. In contrast, a diagnosis of FM is based on one symptom, musculoskeletal pain, which is further detailed through specific WPI items. The specification of musculoskeletal pain in FM may lead to a more interconnected set of symptoms related to pain, potentially overshadowing other symptom relationships in the network. This could create a disproportionate emphasis on pain-related symptoms. Therefore, the network analysis was repeated twice: (1) with the removal of the general FM musculoskeletal pain item, leaving only the more specific symptoms, and (2) with the 19 WPI items being replaced by the WPI sum score (dichotomized into 1 if the total score was ≥ 7 and 0 if the total score was < 7).

| **Supplemental Table 1**. Frequency of diagnostic symptoms of internalizing disorders and functional disorders in females (n = 43,621) and males (n = 29,298) and in adults ≤ 50 years (n = 39,250) and > 50 years (n = 33,669). | | | | | | |
| --- | --- | --- | --- | --- | --- | --- |
| *Symptom* | *% Female* | *% Male* | *χ^2^* | *≤ 50 years (%)* | *> 50 years (%)* | *χ^2^* |
| **MDD** |  |  |  |  |  |  |
| Depressed mood | 4.1 | 3.6 | **14.8** | 4.0 | 3.8 | 2.1 |
| Anhedonia | 5.3 | 5.0 | 4.6 | 5.9 | 4.3 | **90.2** |
| Appetite change | 4.9 | 2.7 | **232.9** | 5.2 | 2.7 | **295.2** |
| Weight increase | 5.0 | 3.5 | **84.1** | 4.8 | 3.9 | **32.4** |
| Weight decrease | 2.2 | 2.0 | 5.4 | 2.4 | 1.8 | **25.6** |
| Psychomotor retardation | 1.2 | 0.9 | **15.1** | 1.0 | 1.1 | 2.1 |
| Psychomotor agitation | 4.9 | 3.7 | **57.3** | 5.3 | 5.3 | **139.0** |
| Guilt | 3.5 | 2.4 | **69.8** | 3.6 | 2.4 | **86.3** |
| Suicidal | 1.0 | 1.2 | 3.8 | 1.2 | 1.0 | 10.7 |
| **GAD** |  |  |  |  |  |  |
| Worry | 7.4 | 4.7 | **211.7** | 7.3 | 5.3 | **121.7** |
| Restless | 28.1 | 11.7 | **586.6** | 17.9 | 13.1 | **308.2** |
| Muscle tension | 28.1 | 19.3 | **720.5** | 27.7 | 20.9 | **453.8** |
| Irritability | 19.1 | 14.3 | **279.1** | 21.5 | 12.1 | **1124.0** |
| **CFS** |  |  |  |  |  |  |
| Sore throat | 1.8 | 1.3 | **22.1** | 1.7 | 1.5 | 3.6 |
| Tender lymph nodes | 1.6 | 0.9 | **65.9** | 1.5 | 1.1 | **21.3** |
| Joint pain | 23.8 | 16.6 | **547.6** | 15.9 | 26.8 | **1299.8** |
| Post exertional malaise | 12.6 | 7.8 | **416.7** | 8.6 | 12.9 | **328.4** |
| Muscle pain | 15.7 | 11.8 | **214.8** | 12.1 | 16.4 | **278.7** |
| Headaches | 9.0 | 4.8 | **459.5** | 8.5 | 6.0 | **169.6** |
| **FM** |  |  |  |  |  |  |
| Musculoskeletal pain | 16.8 | 12.0 | **317.3** | 12.3 | 17.8 | **431.4** |
| Shoulder pain | 34.6 | 26.0 | **611.5** | 30.0 | 32.6 | **67.9** |
| Hip pain | 23.1 | 14.0 | **930.1** | 16.4 | 23.0 | **353.7** |
| Upper arm pain | 19.6 | 12.4 | **665.3** | 13.9 | 19.9 | **485.6** |
| Lower arm pain | 11.5 | 9.2 | **105.5** | 10.1 | 11.1 | **21.3** |
| Upper leg pain | 14.0 | 9.7 | **306.4** | 10.2 | 14.7 | **357.2** |
| Lower leg pain | 13.8 | 12.3 | **30.6** | 11.3 | 15.4 | **284.4** |
| Jaw pain | 6.1 | 3.4 | **277.0** | 5.2 | 4.8 | 3.3 |
| Chest pain | 7.2 | 6.8 | 5.1 | 7.2 | 6.9 | 1.5 |
| Abdomen pain | 18.0 | 7.7 | **1559.7** | 15.7 | 11.6 | **242.07** |
| Upper back pain | 16.6 | 8.1 | **1128.5** | 14.8 | 11.3 | **181.4** |
| Lower back pain | 43.7 | 37.9 | **250.0** | 41.5 | 41.2 | 0.2 |
| Neck pain | 37.8 | 23.8 | **1583.4** | 32.6 | 31.7 | 5.4 |
| Difficulty thinking | 15.7 | 11.7 | **232.3** | 15.4 | 12.5 | **123.4** |
| **IBS** |  |  |  |  |  |  |
| IBS symptom | 10.6 | 4.7 | **803.3** | 9.2 | 7.0 | **116.3** |
| **Overlapping Symptoms** |  |  |  |  |  |  |
| Trouble sleeping | 36.6 | 24.0 | **1274.6** | 29.5 | 33.9 | **162.4** |
| Unrefreshing sleep | 41.4 | 32.7 | **567.0** | 42.2 | 32.9 | **663.6** |
| Fatigue | 44.0 | 31.8 | **1098.3** | 45.2 | 32.1 | **1285.3** |
| Difficulty concentrating | 34.8 | 29.2 | **254.5** | 33.2 | 31.8 | 13.3 |

Differences in symptom frequency for each symptom were compared with the chi-square (ꭓ^2^). Values in bold are significantly different between groups at Bonferroni corrected α = 0.001. MDD: major depressive disorder. GAD: generalized anxiety disorder. CFS: chronic fatigue syndrome. FM: fibromyalgia. IBS: irritable bowel syndrome.


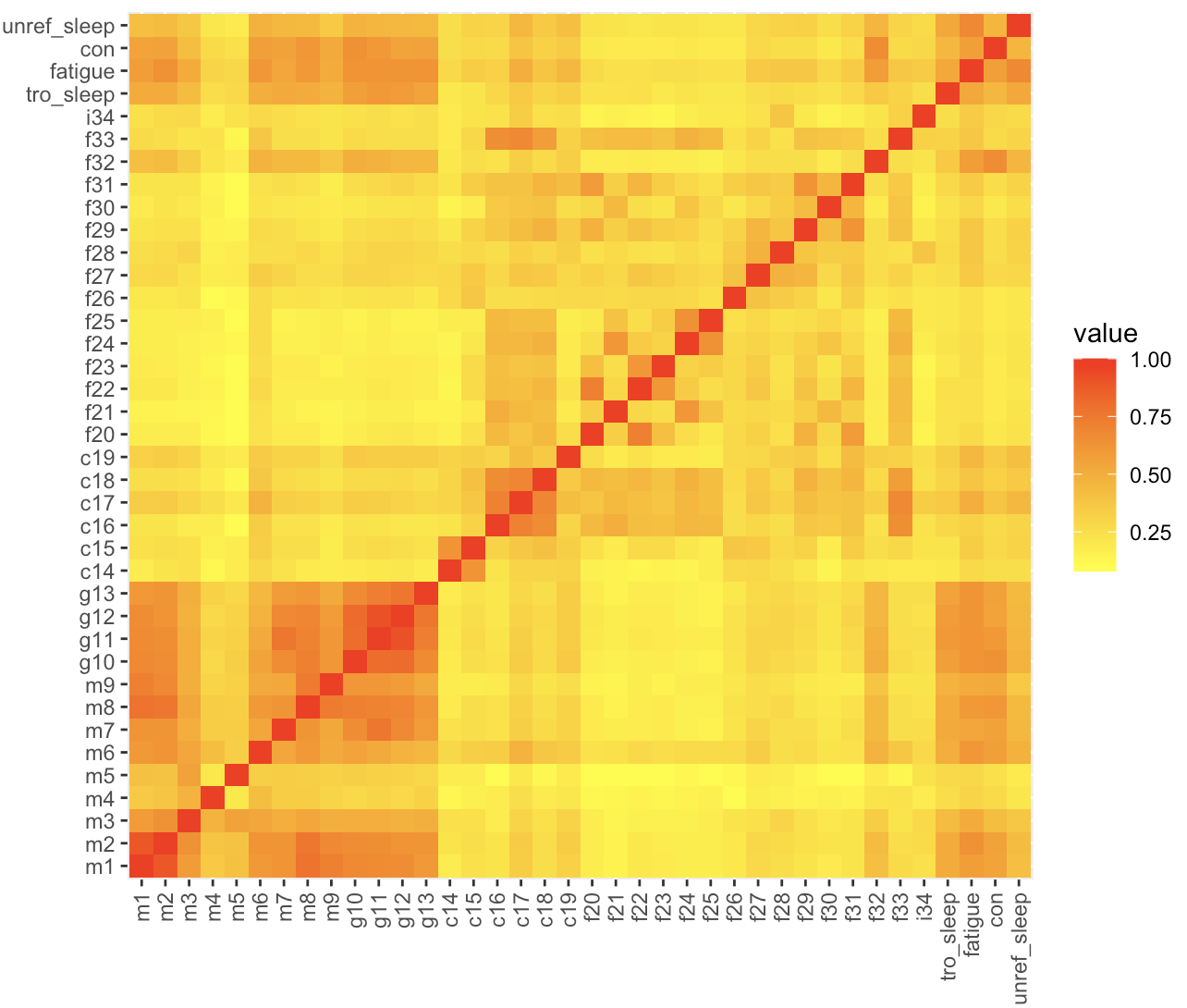


**Supplemental Figure 1**. **Tetrachoric correlation heatmap of ID and FD symptoms**. MDD, major depressive disorder; GAD, generalized anxiety disorder; CFS, chronic fatigue syndrome; WPI, widespread pain index; CIS, checklist individual strength; FM, fibromyalgia; IBS, irritable bowel syndrome. WPI and CIS belong to the FM symptoms. m1 = depressed mood; m2 = anhedonia; m3 = appetite change; m4 = weight gain; m5 = weight loss; m6 = psychomotor retardation; m7 = psychomotor agitation; m8 = guilt; m9 = suicidal; g10 = worry; g11 = restless; g12 = muscle tension; g13 = irritable; c14 = sore throat; c15 = tender lymph nodes; c16 = joint pain; c17 = post-exertional malaise; c18 = headaches; c19 = muscle pain; f20 = WPI shoulder pain; f21 = WPI hip pain; f22 = WPI upper arm pain; f23 = WPI lower arm pain; f24 = WPI upper leg pain; f25 = WPI lower leg pain; f26 = WPI jaw pain; f27 = WPI chest pain; f28 = WPI abdomen pain; f29 = WPI upper back pain; f30 = WPI lower back pain; f31 = WPI neck pain; f32 = CIS difficulty thinking; f33 = musculoskeletal pain; i34 = IBS symptom; tro_sleep = trouble sleeping; con = concentration; unref_sleep = unrefreshing sleep.


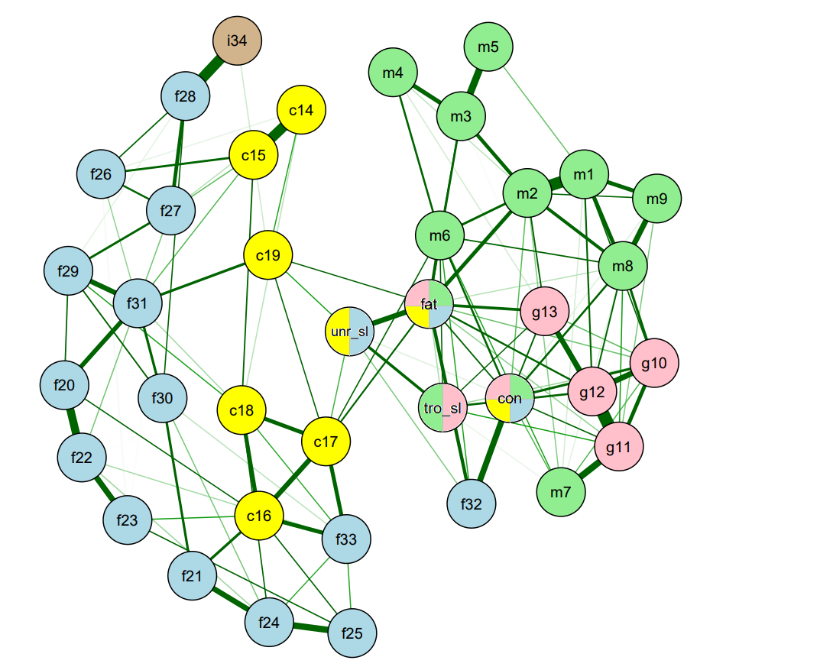

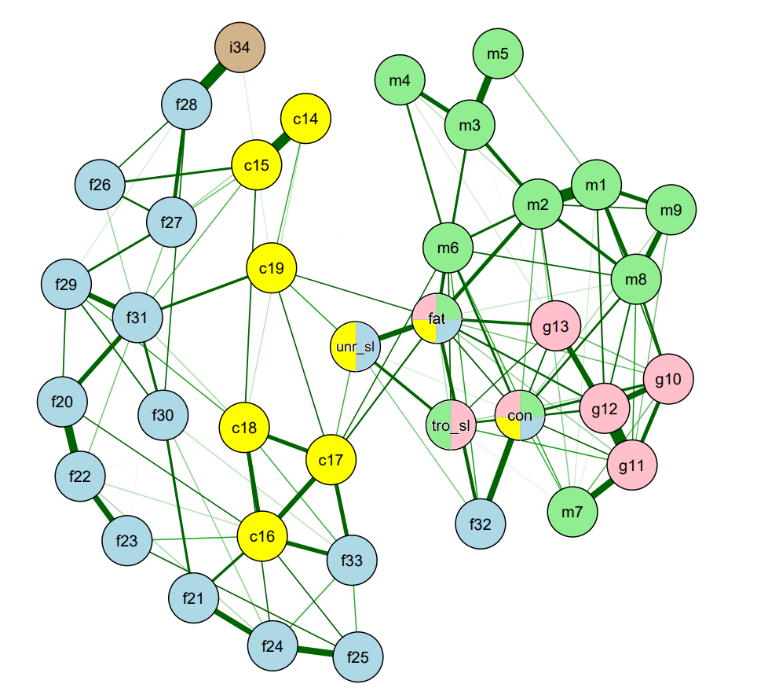


2. nd = 0.51

1. nd = 0.51


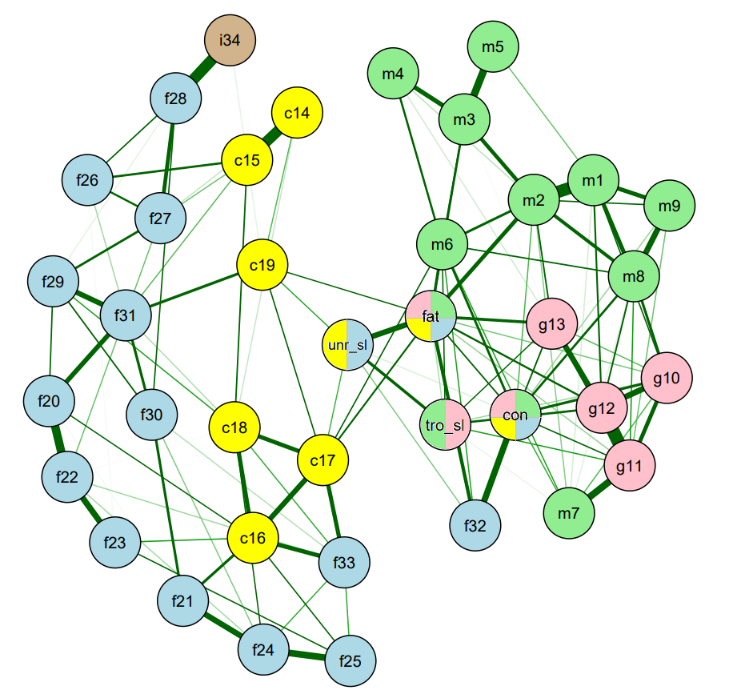


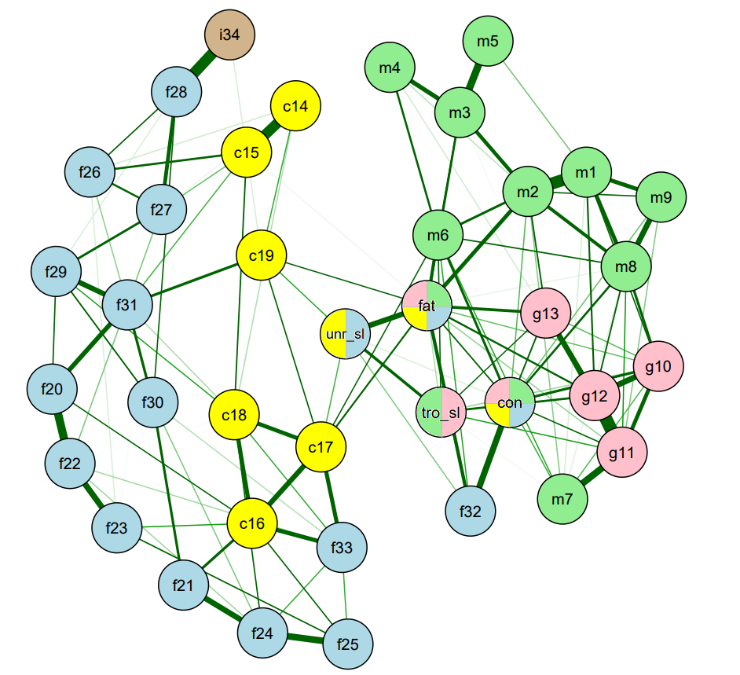


4. nd = 0.50

3. nd = 0.51


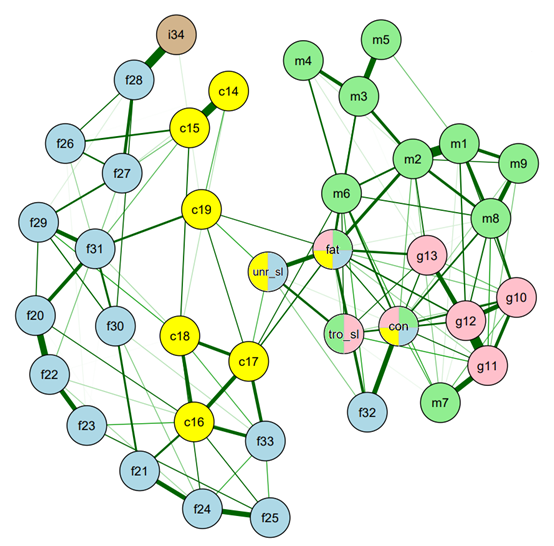

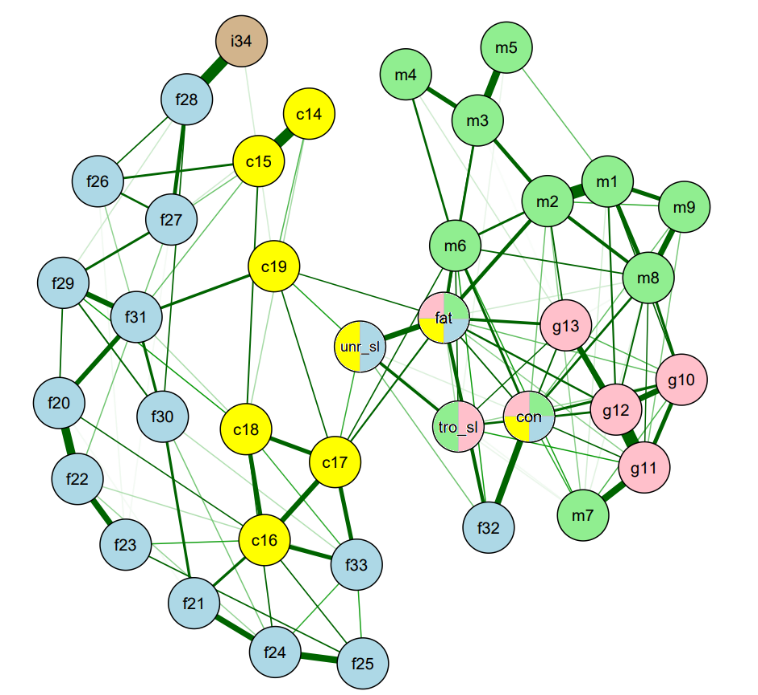


6. Aggregated network

5. nd = 0.51

**Supplemental Figure 2**. Full network as estimated with eLasso, in 72,919 individuals of the five imputed datasets. Network nr. 6 is the aggregated network presented in the main text. Green lines indicate positive edges. Line thickness and color intensity display edge strength. Only edges with weight ≥ 0.3 are presented. Networks were fitted using the R package *IsingFit* (van Borkulo et al., 2016) and network plots were generated using the *qgraph* package (Epskamp et al., 2012). Green nodes = MDD; pink nodes = GAD; yellow nodes = CFS; blue nodes = FM; brown node = IBS. Symptom coding is same as in Figure 1 in main text.


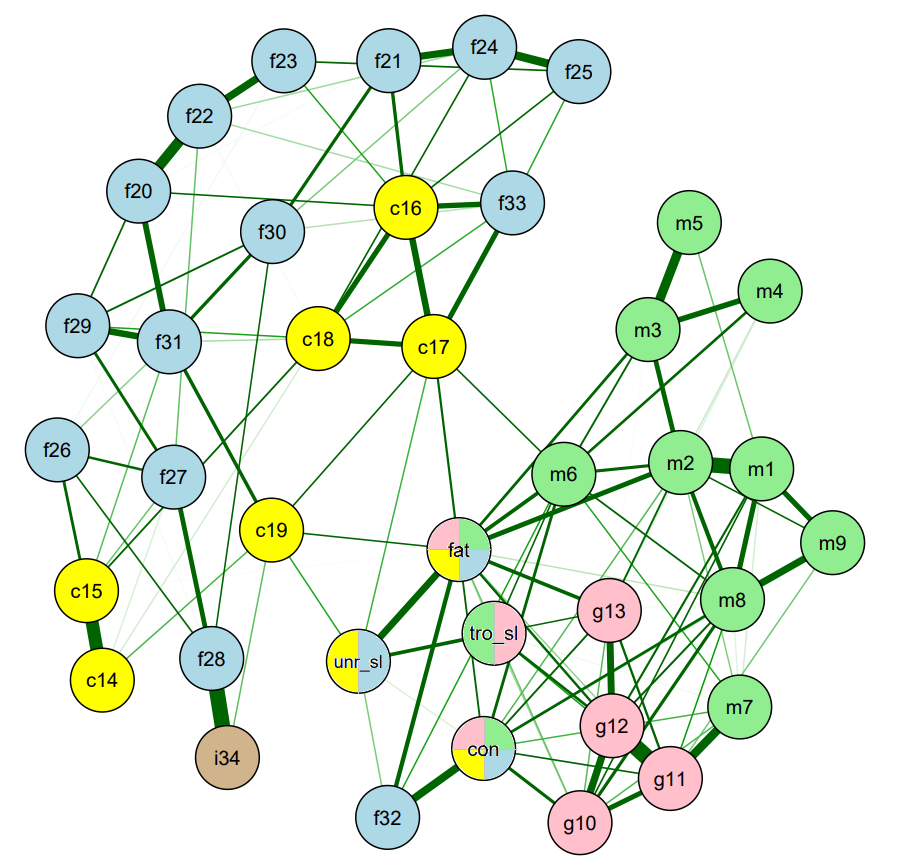


**Supplemental Figure 3**. Full network as estimated with eLasso for the IBS sensitivity analysis. Green lines indicate positive edges. Line thickness and color intensity display edge strength. Only edges with weight ≥ 0.3 are presented. Networks were fitted using the R package *IsingFit* (van Borkulo et al., 2016) and network plots were generated using the *qgraph* (Epskamp et al., 2012) package. Green nodes = MDD; pink nodes = GAD; yellow nodes = CFS; blue nodes = FM; brown node = IBS. Symptom coding is same as in Figure 1 in main text.


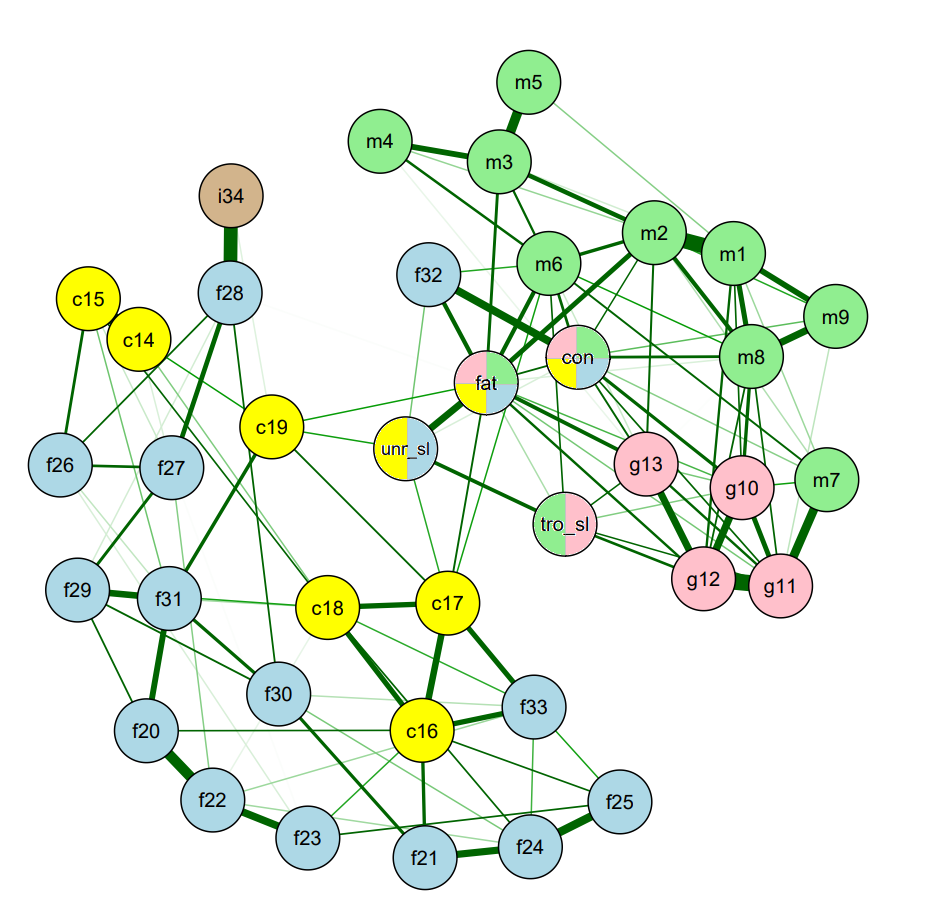


**Supplemental Figure 4**. Full network as estimated with eLasso for the other medical conditions sensitivity analysis. Green lines indicate positive edges. Line thickness and color intensity display edge strength. Only edges with weight ≥ 0.3 are presented. Networks were fitted using the R package *IsingFit* (van Borkulo et al., 2016) and network plots were generated using the *qgraph* (Epskamp et al., 2012) package. Green nodes = MDD; pink nodes = GAD; yellow nodes = CFS; blue nodes = FM; brown node = IBS. Symptom coding is same as in Figure 1 in main text.


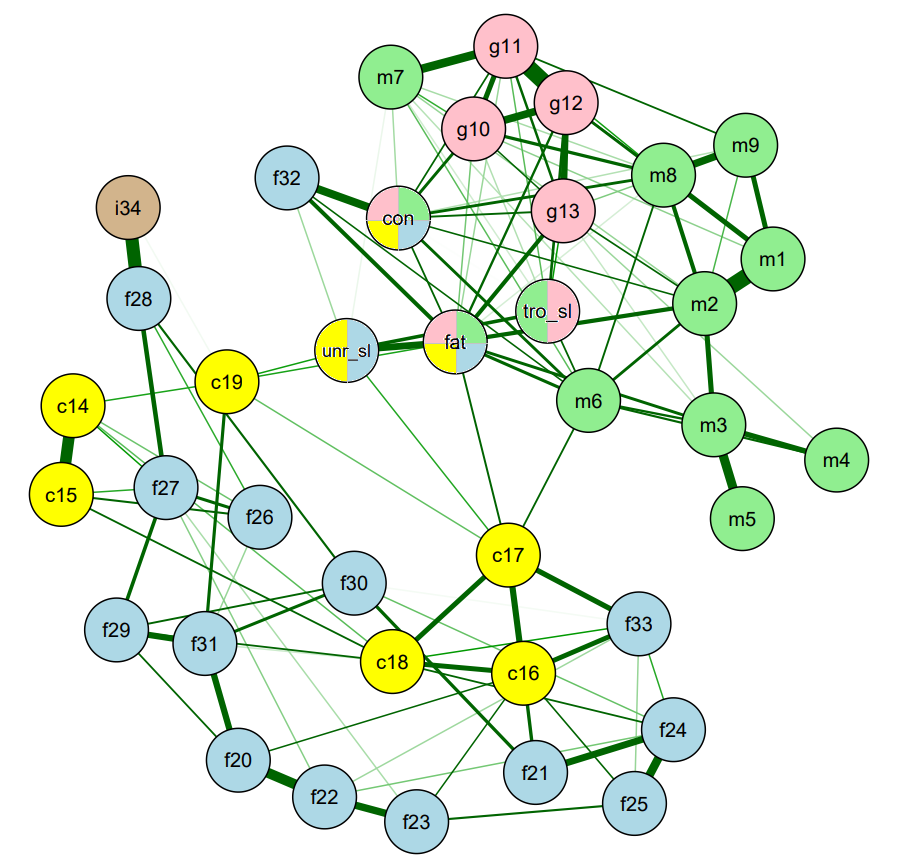

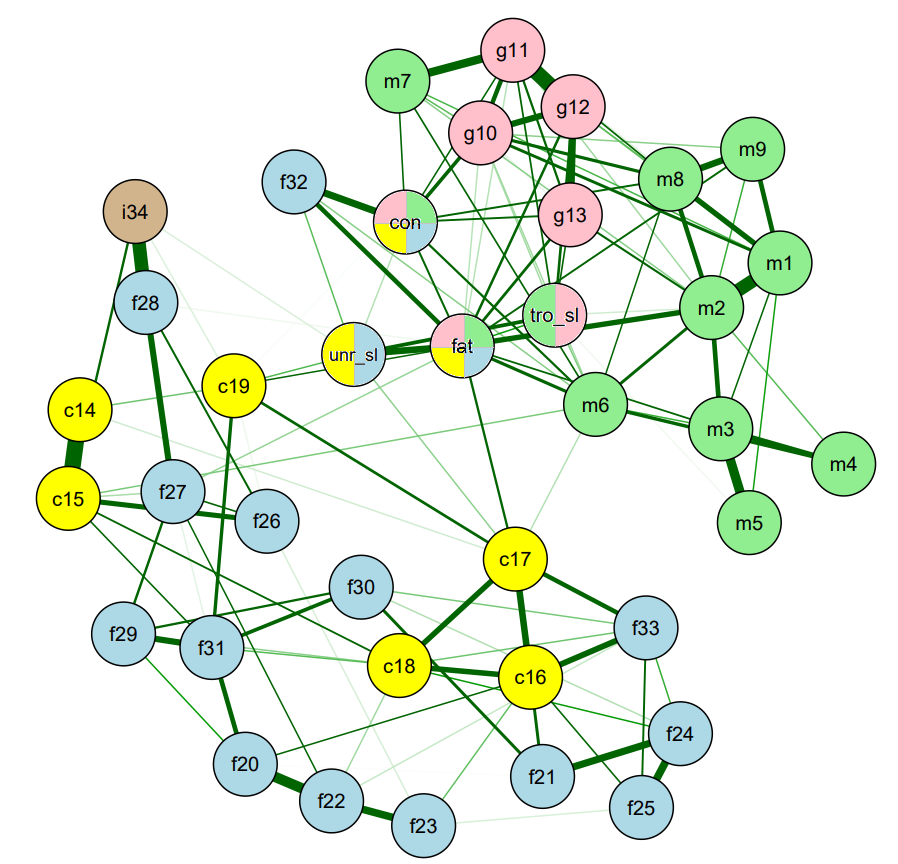


1. Female subjects
2. Male subjects

**Supplemental Figure 5**. Full network as estimated with eLasso, in 43,621 females and 29,298 males. Green lines indicate positive edges. Line thickness and color intensity display edge strength. Only edges with weight ≥ 0.3 are presented. Networks were fitted using the R package *IsingFit* (van Borkulo et al., 2016) and network plots were generated using the *qgraph* (Epskamp et al., 2012) package. Green nodes = MDD; pink nodes = GAD; yellow nodes = CFS; blue nodes = FM; brown node = IBS. Symptom coding is same as in Figure 1 in main text.

| **Supplemental Table 2.** NCT results | | | | | |
| --- | --- | --- | --- | --- | --- |
| **Male vs female** | | | **Older vs younger adults** | | |
| Imputed dataset | Network invariance | Global strength | Imputed dataset | Network invariance | Global strength |
| 1 | 0.04* | 0.95 | 1 | 0.01* | 0.61 |
| 2 | 0.05* | 0.98 | 2 | 0.01* | 0.33 |
| 3 | 0.03* | 0.97 | 3 | 0.01* | 0.88 |
| 4 | 0.06 | 0.95 | 4 | 0.01* | 0.75 |
| 5 | 0.03* | 0.93 | 5 | 0.01* | 0.94 |

Displayed are the *p*-values of the network invariance and global strength of the five imputed datasets. **P*-values are considered significant at an alpha of 0.05. Edge strengths were not tested if no robust differences in network structures between subgroups were found.

| **Supplemental Table 3.** Sensitivity analysis with balanced equal sample sizes for the sex subgroups | | | | | | | | |
| --- | --- | --- | --- | --- | --- | --- | --- | --- |
| Imputed data 1 | Network invariance | Global strength | Imputed data 3 | Network invariance | Global strength | Imputed data 5 | Network structure | Global strength |
| 1 | 0.08 | 0.12 | 1 | 0.17 | 0.00* | 1 | 0.15 | 0.07 |
| 2 | 0.10 | 0.06 | 2 | 0.11 | 0.03* | 2 | 0.06 | 0.00* |
| 3 | 0.02* | 0.01* | 3 | 0.04* | 0.01* | 3 | 0.02* | 0.00* |
| 4 | 0.00* | 0.04* | 4 | 0.12 | 0.00* | 4 | 0.03* | 0.02* |
| 5 | 0.07 | 0.06 | 5 | 0.02* | 0.04* | 5 | 0.09 | 0.07 |
| 6 | 0.06 | 0.23 | 6 | 0.05* | 0.04* | 6 | 0.04* | 0.12 |
| 7 | 0.13 | 0.01* | 7 | 0.05* | 0.00* | 7 | 0.06 | 0.05* |
| 8 | 0.22 | 0.00* | 8 | 0.11 | 0.01* | 8 | 0.24 | 0.03* |
| 9 | 0.01* | 0.05* | 9 | 0.10 | 0.00* | 9 | 0.17 | 0.36 |
| 10 | 0.06 | 0.10 | 10 | 0.02* | 0.08 | 10 | 0.07 | 0.00* |
| Imputed data 2 | Network invariance | Global strength | Imputed data 4 | Network invariance | Global strength |  |  |  |
| 1 | 0.09 | 0.08 | 1 | 0.07 | 0.28 |  |  |  |
| 2 | 0.01* | 0.00* | 2 | 0.12 | 0.34 |  |  |  |
| 3 | 0.12 | 0.01* | 3 | 0.13 | 0.04* |  |  |  |
| 4 | 0.04* | 0.01* | 4 | 0.19 | 0.74 |  |  |  |
| 5 | 0.03* | 0.06 | 5 | 0.03* | 0.21 |  |  |  |
| 6 | 0.20 | 0.00* | 6 | 0.07 | 0.33 |  |  |  |
| 7 | 0.07 | 0.00* | 7 | 0.14 | 0.02* |  |  |  |
| 8 | 0.07 | 0.01* | 8 | 0.05* | 0.15 |  |  |  |
| 9 | 0.35 | 0.12 | 9 | 0.22 | 0.31 |  |  |  |
| 10 | 0.22 | 0.02* | 10 | 0.06 | 0.11 |  |  |  |

Displayed are the *p*-values of the network invariance and global strength of the 10 subsamples from the five imputed datasets. **P*-values are considered significant at an alpha of 0.05.


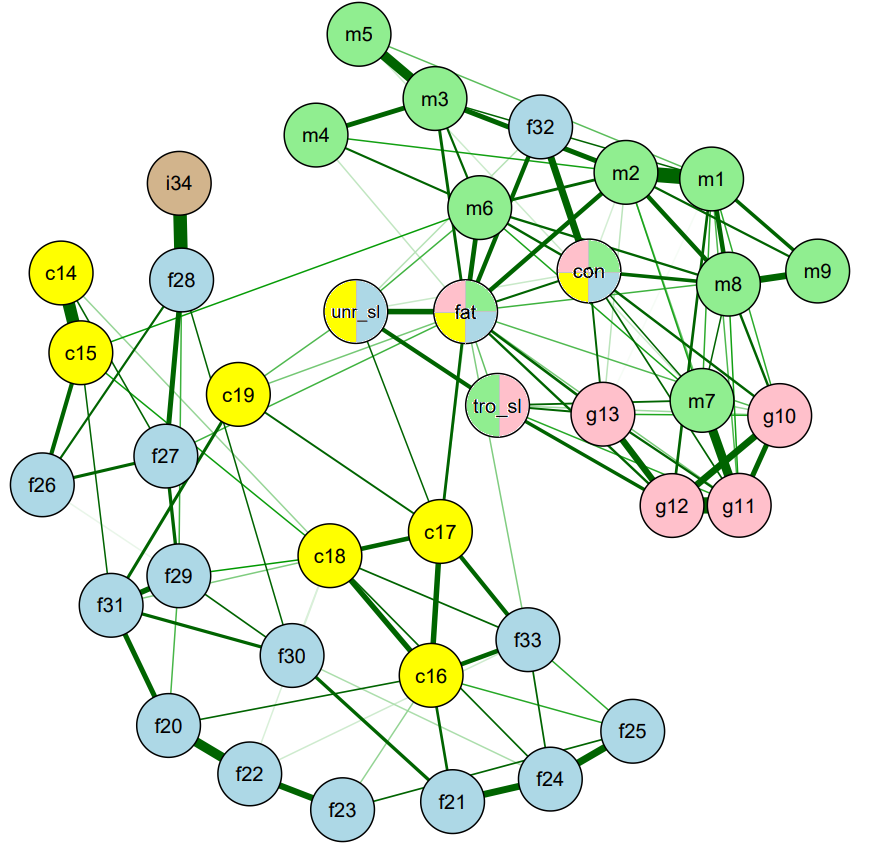

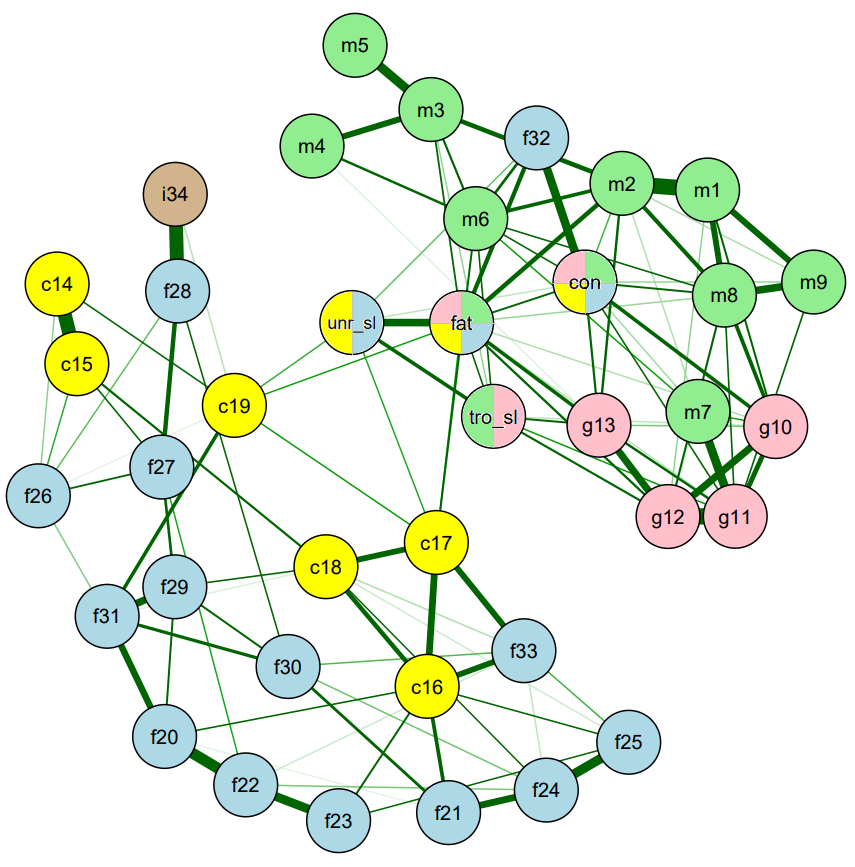


**Supplemental Figure 6**. Full network as estimated with eLasso, in 39,250 subjects 50 years old or younger and 33,669 subjects older than 50 years. Green lines indicate positive edges. Line thickness and color intensity display edge strength. Only edges with weight ≥ 0.3 are presented. Networks were fitted using the R package *IsingFit* (van Borkulo et al., 2016) and network plots were generated using the *qgraph* package (Epskamp et al., 2012). Green nodes = MDD; pink nodes = GAD; yellow nodes = CFS; blue nodes = FM; brown node = IBS. Symptom coding is same as in Figure 1 in main text.

(b) Subjects > 50 years old

1. Subjects ≤ 50 years

| **Supplemental Table 4.** Sensitivity analysis with balanced subsamples for the age subgroups ≤ 50 and > 50. | | | | | | | | |
| --- | --- | --- | --- | --- | --- | --- | --- | --- |
| Imputed data 1 | Network invariance | Global strength | Imputed data 3 | Network invariance | Global strength | Imputed data 5 | Network structure | Global strength |
| 1 | 0.00 | 0.24 | 1 | 0.17 | 0.00* | 1 | 0.01* | 0.10 |
| 2 | 0.10 | 0.05* | 2 | 0.11 | 0.03* | 2 | 0.00* | 0.37 |
| 3 | 0.00* | 0.75 | 3 | 0.04* | 0.01* | 3 | 0.00* | 0.73 |
| 4 | 0.01* | 0.38 | 4 | 0.12 | 0.00* | 4 | 0.00* | 0.20 |
| 5 | 0.03* | 0.16 | 5 | 0.02* | 0.04* | 5 | 0.01* | 0.35 |
| 6 | 0.06 | 0.18 | 6 | 0.05* | 0.04* | 6 | 0.02* | 0.38 |
| 7 | 0.00* | 0.16 | 7 | 0.05* | 0.00* | 7 | 0.00* | 0.41 |
| 8 | 0.17 | 0.77 | 8 | 0.11 | 0.01* | 8 | 0.22 | 0.83 |
| 9 | 0.30 | 0.36 | 9 | 0.10 | 0.00* | 9 | 0.00* | 0.43 |
| 10 | 0.06 | 0.10 | 10 | 0.02* | 0.08 | 10 | 0.00* | 0.39 |
| Imputed data 2 | Network invariance | Global strength | Imputed data 4 | Network invariance | Global strength |  |  |  |
| 1 | 0.09 | 0.08 | 1 | 0.00* | 0.12 |  |  |  |
| 2 | 0.01* | 0.00* | 2 | 0.09 | 0.70 |  |  |  |
| 3 | 0.12 | 0.01* | 3 | 0.14 | 0.90 |  |  |  |
| 4 | 0.04* | 0.01* | 4 | 0.08 | 0.29 |  |  |  |
| 5 | 0.03* | 0.06 | 5 | 0.00* | 0.23 |  |  |  |
| 6 | 0.20 | 0.00* | 6 | 0.00* | 0.09 |  |  |  |
| 7 | 0.07 | 0.00* | 7 | 0.00* | 0.15 |  |  |  |
| 8 | 0.07 | 0.01* | 8 | 0.03* | 0.84 |  |  |  |
| 9 | 0.35 | 0.12 | 9 | 0.00* | 0.50 |  |  |  |
| 10 | 0.22 | 0.02* | 10 | 0.00* | 0.79 |  |  |  |

Displayed are the *p*-values of the network invariance and global strength of the 10 subsamples from the five imputed datasets. *P*-values are considered significant at an alpha of 0.05.


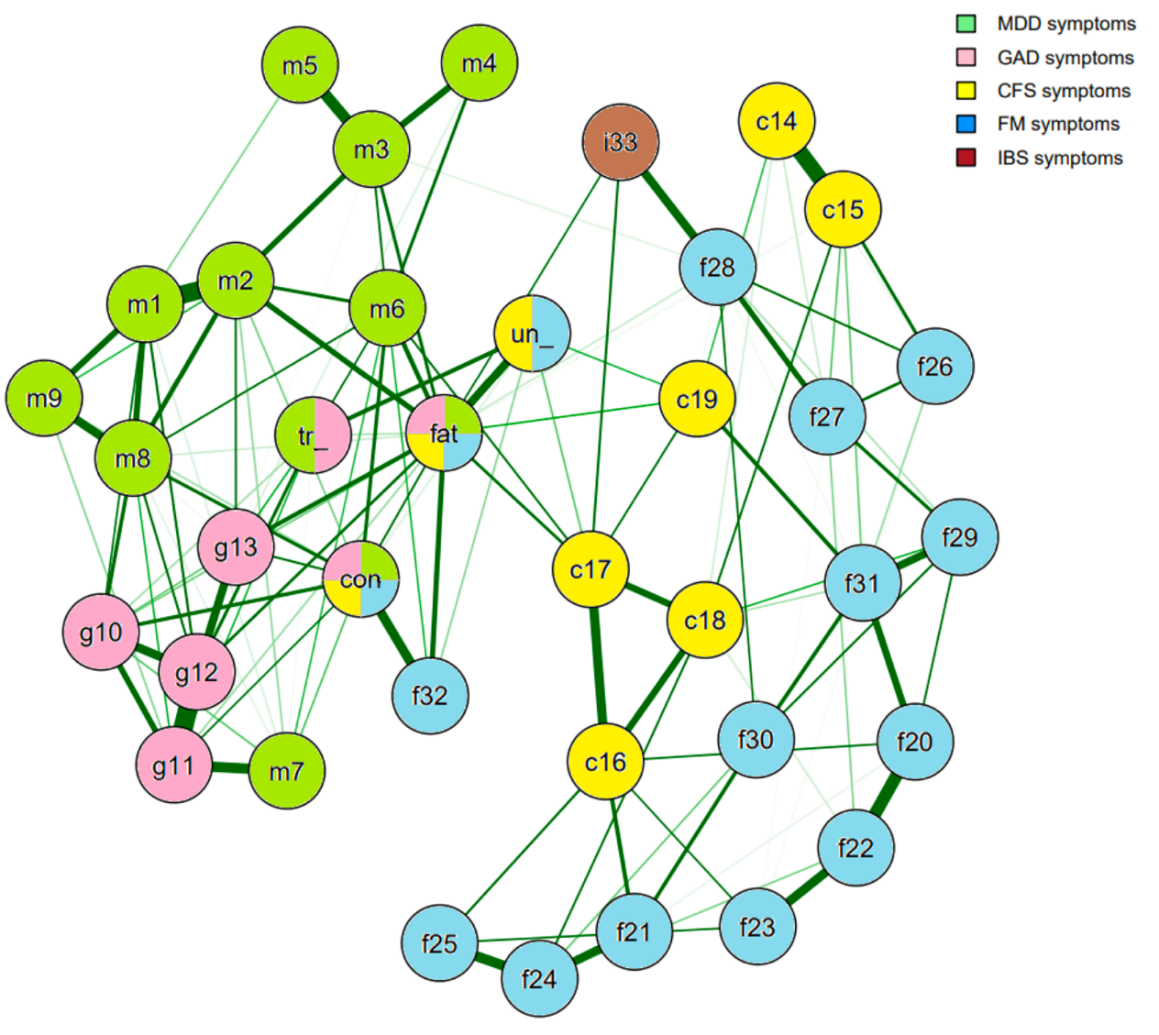


**Supplemental Figure 7.** Network with the FM symptom, musculoskeletal pain, symptom removed. m1 = depressed mood; m2 = anhedonia; m3 = appetite change; m4 = weight gain; m5 = weight loss; m6 = retardation; m7 = agitation; m8 = guilt; m9 = suicidal; g10 = worry; g11 = restless; g12 = tense; g13 = irritability; c14 = sore throat; c15 = tender lymph nodes; c16 = joint pain; c17 = post-exertional malaise; c18 = muscle pain; c19 = headaches; f20 = WPI shoulder; f21 = WPI hip; f22 = WPI upper arm; f23 = WPI lower arm; f24 = WPI upper leg; f25 = WPI lower leg; f26 = WPI jaw; f27 = WPI chest; f28 = WPI abdomen; f29 = WPI upper back; f30 = WPI lower back; f31 = WPI neck; f32 = CIS difficulty thinking; i33 = IBS abdominal pain; tro_sl = trouble sleeping; fat = fatigue; con = concentration; unr_sl = unrefreshing sleep.


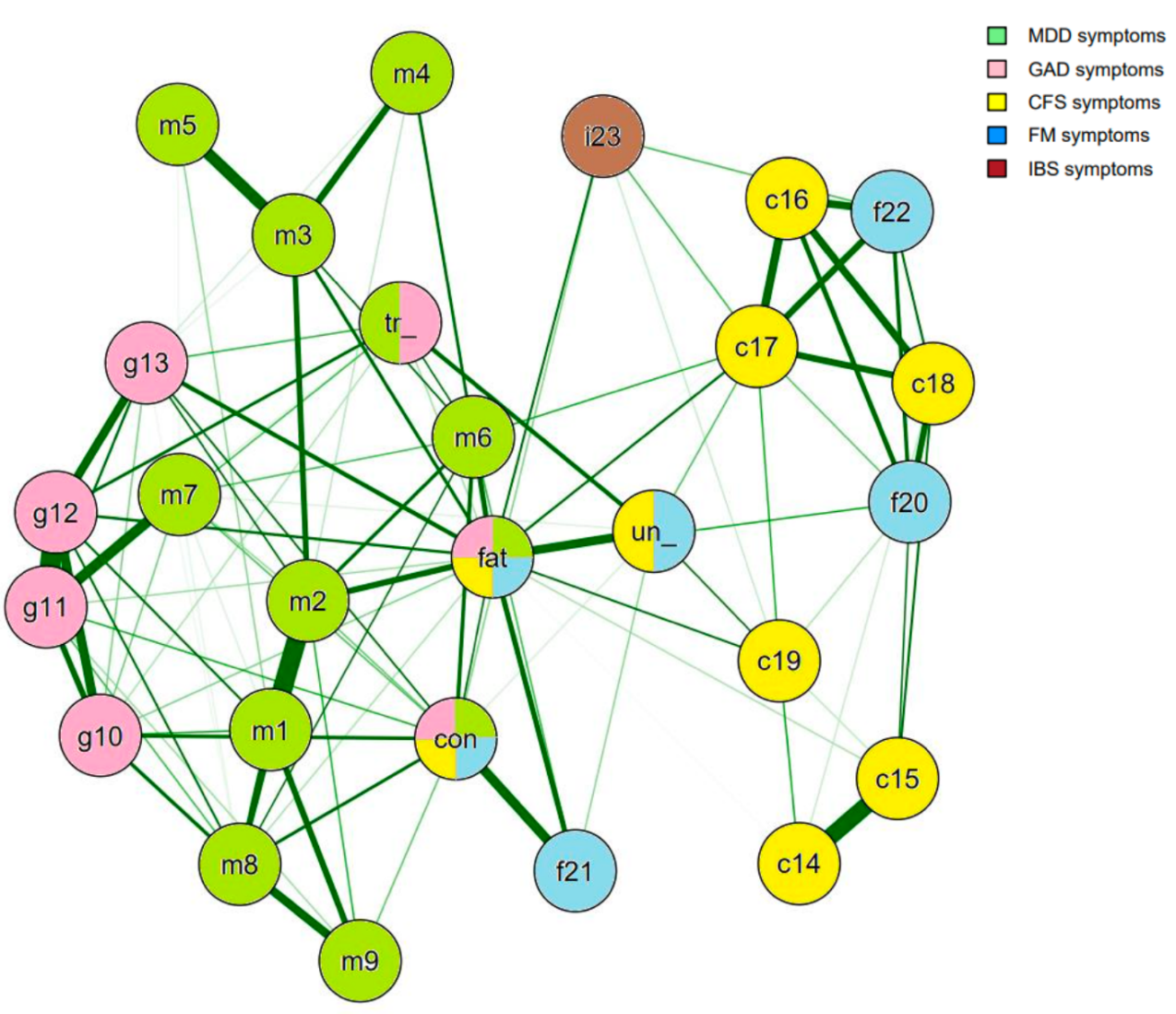


**Supplemental Figure 8.** Network with WPI items combined into one symptom. m1 = depressed mood; m2 = anhedonia; m3 = appetite change; m4 = weight gain; m5 = weight loss; m6 = retardation; m7 = agitation; m8 = guilt; m9 = suicidal; g10 = worry; g11 = restless; g12 = tense; g13 = irritability; c14 = sore throat; c15 = tender lymph nodes; c16 = joint pain; c17 = post-exertional malaise; c18 = muscle pain; c19 = headaches; f20 = WPI summed; f21 = CIS difficulty thinking; f22 = musculoskeletal pain; i23 = IBS abdominal pain; tro_sl = trouble sleeping; fat = fatigue; con = concentration; unr_sl = unrefreshing sleep.

**References**

[1] Arrindel, W. A., & Ettema, J. H. M. (2003). Handleiding bij een multidimensionele psychopathologie-indicator. Symptom Checklist SCL-90.(Herziene uitgave)[Manual for a multidimensional psychopathology-indicator. Symptom Checklist SCL-90 (revised edition)].

[2] Borsboom, D., Deserno, M. K., Rhemtulla, M., Epskamp, S., Fried, E. I., McNally, R. J., & Waldorp, L. J. (2021). Network analysis of multivariate data in psychological science. *Nature Reviews Methods Primers*, *1*(1), 58. https://doi.org/10.1038/s43586-021-00060-z.
